# Dr. Archibald Mc M. Dann

**Published:** 1914-10

**Authors:** 


Dr. Archibald Mc M. Dann, Pennsylvania, 1866, died at his home in Rochester in September. He was born February 3, 1845 in Rush, and was educated at the Genesee Wesleyan Seminary of Lima and Middlebury Academy, Wyoming. After graduation, he practiced in Honeoye Falls till 1872, when he removed to Rochester. He had been president of the Monroe County Society and the Rochester Pathologic Society, had served on the staff of the Rochester General Hospital and was Consultant to the Rochester State Hospital. He was a member of the Doctors Club.

